# Cord blood *Streptococcus pneumoniae‐*specific cellular immune responses predict early pneumococcal carriage in high‐risk infants in Papua New Guinea

**DOI:** 10.1111/cei.12902

**Published:** 2016-12-18

**Authors:** J. P. Francis, P. C. Richmond, D. Strickland, S. L. Prescott, W. S. Pomat, A. Michael, M. A. Nadal‐Sims, C. J. Edwards‐Devitt, P. G. Holt, D. Lehmann, A. H. J. van den Biggelaar

**Affiliations:** ^1^ Papua New Guinea Institute of Medical ResearchGorokaPapua New Guinea; ^2^ School of Paediatrics and Child HealthUniversity of Western AustraliaPerthAustralia; ^3^ Telethon Kids Institute, University of Western AustraliaPerthAustralia

**Keywords:** colonization, newborn, Papua New Guinea, pneumococcal immunity, PspA

## Abstract

In areas where *Streptococcus pneumoniae* is highly endemic, infants experience very early pneumococcal colonization of the upper respiratory tract, with carriage often persisting into adulthood. We aimed to explore whether newborns in high‐risk areas have pre‐existing pneumococcal‐specific cellular immune responses that may affect early pneumococcal acquisition. Cord blood mononuclear cells (CBMC) of 84 Papua New Guinean (PNG; high endemic) and 33 Australian (AUS; low endemic) newborns were stimulated *in vitro* with detoxified pneumolysin (dPly) or pneumococcal surface protein A (PspA; families 1 and 2) and compared for cytokine responses. Within the PNG cohort, associations between CBMC dPly and PspA‐induced responses and pneumococcal colonization within the first month of life were studied. Significantly higher PspA‐specific interferon (IFN)‐γ, tumour necrosis factor (TNF)‐α, interleukin (IL)‐5, IL‐6, IL‐10 and IL‐13 responses, and lower dPly‐IL‐6 responses were produced in CBMC cultures of PNG compared to AUS newborns. Higher CBMC PspA‐IL‐5 and PspA‐IL‐13 responses correlated with a higher proportion of cord CD4 T cells, and higher dPly‐IL‐6 responses with a higher frequency of cord antigen‐presenting cells. In the PNG cohort, higher PspA‐specific IL‐5 and IL‐6 CBMC responses were associated independently and significantly with increased risk of earlier pneumococcal colonization, while a significant protective effect was found for higher PspA‐IL‐10 CBMC responses. Pneumococcus‐specific cellular immune responses differ between children born in pneumococcal high *versus* low endemic settings, which may contribute to the higher risk of infants in high endemic settings for early pneumococcal colonization, and hence disease.

## Introduction

*Streptococcus pneumoniae* remains a leading cause of death in children < 5 years of age, causing more than 500 000 deaths and nearly 14 million episodes of disease annually 1. The spectrum of pneumococcal disease varies from mild‐to‐moderate mucosal disease (acute otitis media, sinusitis, non‐bacteraemic pneumonia) to severe invasive disease (sepsis, meningitis, bacteraemic pneumonia). Most cases of pneumococcal‐associated deaths and severe disease occur in low‐income countries in children < 6 months of age 1, 2. Nasopharyngeal colonization is an immediate precursor to disease 3, 4. In low‐income settings infants become colonized rapidly, with carriage often persisting into adulthood 5. Early onset of pneumococcal carriage is considered an important risk factor for the high burden of serious pneumococcal disease and death in young infants 6, 7, 8, 9.

The decreasing incidence of invasive pneumococcal disease (IPD) with increasing age implies the acquisition of protective immunity over time. While serotype‐specific antibodies against capsular polysaccharides are a main mechanism of immune protection, a number of conserved pneumococcal proteins with immunogenic properties have been identified, including detoxified pneumolysin (dPly) and pneumococcal surface protein A (PspA) 10, 11, 12. Pneumolysin is a cytolysin produced by *S. pneumoniae* that enables the bacteria to penetrate the host's physical defences through its cytotoxic effect on epithelial cells, thus facilitating carriage and disease 13. PspA is a cell wall‐associated protein that plays a role in inhibiting complement‐mediated opsonization 14, 15 and can prevent lactoferrin‐mediated clearance 16. Animal models have demonstrated that pneumococcal colonization and infection result in the induction of cellular and humoral immune responses to both dPly and PspA 17, 18. We reported recently that in young infants in Papua New Guinea (PNG), a country highly endemic for pneumococcal infections, PspA‐specific antibodies are high at the time of birth and decline during the first 3 months of life, presumably due to waning of maternal antibodies, followed by an increase with ongoing exposure and increasing age 19. In this same study population, higher levels of naturally acquired immunoglobulin (Ig)G antibodies to dPly at birth were associated with delayed pneumococcal colonization during the first month of life; however, the opposite was observed for cord PspA‐specific antibodies 5. A US study, including 11 adults and 17 children (aged 6 months to 3 years), showed that *in‐vitro* responses to pneumococcal proteins, including PspA and dPly, were dominated by T helper type 1 (Th1) responses [interferon (IFN)‐γ, interleukin (IL)‐2] or primed ‘uncommitted’ (IL‐2) responses in adults, while in children responses were mainly ‘uncommitted’ IL‐2 responses 20. An earlier study conducted by Zhang *et al*. in the United Kingdom reported that CD4 T cells (recombinant) pneumolysin‐induced proliferation and IFN‐γ and tumour necrosis factor (TNF)‐α production were reduced if children (aged 3–10 years) were pneumococcal carriers 21. We reported earlier that in our PNG cohort very early acquisition of pneumococcal colonization (within the first 2 weeks of life) was associated with impaired development of peripheral blood cellular IFN‐γ and IL‐10 responses to dPly in infancy 22. These observations suggest that the development of pneumococcal‐specific cellular immune responses may vary as a result of the density and age at which pneumococcal exposure is experienced in childhood depending on living in a low‐ *versus* high‐endemic setting; this is in line with the hypothesis that the development of protective T cell responses is compromised when highly exposed at a younger age 23.

In the Eastern Highlands Province (EHP) of PNG, infants are colonized with *S. pneumoniae* within the first 3 months of life, with a median age of pneumococcal acquisition of 17–18 days 5, 24. In comparison, in a high‐income country such as Australia, fewer than half of non‐Indigenous Australian children carry pneumococci at least once during their first year of life 25. The aim of this study was to test the hypothesis that pneumococcal cellular immune responses at birth differ between children born in pneumococcal high‐ *versus* low pneumococcal‐endemic settings, possibly as a result of prenatal activation, and influence infants' subsequent susceptibility to pneumococcal acquisition. Cord blood mononuclear cells (CBMC) from infants born in PNG (high‐endemic) and Australia (AUS; low‐endemic) were stimulated *in vitro* with the pneumococcal proteins dPly or PspA and cytokine responses were compared to identify differences in pneumococcal‐specific cellular immune responses. Next, we studied within the PNG cohort associations between CBMC pneumococcal‐specific immune responses and early pneumococcal acquisition.

## Materials and methods

### Study population

Pregnant women in the Asaro Valley (EHP, PNG) were recruited as part of a Neonatal Pneumococcal Conjugate Vaccine (PCV) Trial conducted between 2005 and 2009 (ClinicalTrials.gov number NCT00219401) 26, 27. Of the 312 children enrolled into that trial, data from 84 children were included in this study, based on the availability of CBMC. As part of the vaccination trial, PNG infants were followed for the acquisition of pneumococcal colonization in the first month of life. In Australia, a cohort of pregnant women representing the general population were recruited through private and state antenatal clinics in the metropolitan area of Perth and CBMC of 33 newborns were included in the current analysis

### Cord blood mononuclear cells (CBMC) isolation

Identical protocols and consumables were used in both settings. Cord blood samples (20–50 ml) were collected in sterile tubes (Sarstedt AG, Nümbrecht, Germany) containing 20 ml of RPMI‐1640 (Invitrogen‐Life Technologies, Melbourne, Australia) and 20 IU/ml of preservative‐free heparin (Pfizer, West Ryde, NSW, Australia). Samples were processed within 18 h from the time of collection to isolate cord blood mononuclear cells (CBMC) by centrifuging twice over a Ficoll‐Hypaque gradient (Lymphoprep, Alexis‐Shield, Oslo, Norway). Cells were cryopreserved at concentrations of 20–50 × 10^6^ cells/ml in 50% heat‐inactivated (HI) fetal calf serum (FCS) (JRH BioSciences, Lenexa, KS, USA) and 7·5% dimethyl sulphoxide. Cells from PNG newborns were transported in a dry‐shipper to Perth for further experiments.

### Flow cytometry

CBMC of 33 Australian newborns and 39 PNG newborns were studied for CD4 T cell frequencies using flow cytometry. Cells expressing high levels of CD4 were considered to be CD4 T cells, while cells expressing low CD4 levels were considered antigen‐presenting cells (APCs). Briefly, CBMC were resuspended in fluorescence‐activated cell sorting (FACS) buffer (1 × 10^6^ cells/tube), incubated on ice with 50 μl of antibodies for CD4 (BD Biosciences, San Jose, CA, USA) (diluted 1 : 10), washed, and then fixed as per the manufacturer's instructions. Data were acquired using a four‐colour FACSCalibur (BD Biosciences) and results analysed using FlowJo software (Tree Star Inc., Stanford, CA, USA). Dead cells were excluded from analysis using a forward‐ *versus* side‐scatter gate.

### Pneumococcal protein antigens

PspA was generated in the laboratory of Professor Wayne Thomas [Division of Molecular Biotechnology, Telethon Kids Institute (former Institute of Child Health Research), Perth, Australia]. The production of the recombinant PspA family 1 (PspA1) and PspA family 2 (PspA2) antigens from *S. pneumoniae* is outlined in Francis *et al*. 5. Briefly, PspA1 (family 1, clade 2) was derived from the recombinant PspA/Rx1_AA1…302_ protein, which comprised 302 N‐terminal amino acids of the pneumococcal strain Rx1 PspA, and PspA2 (family 2, clade 3) from PspA/V‐24_AA1…410_, consisting of amino acids 1–410 of the wild‐type mature PspA *S. pneumoniae* Taiwan19F‐14. Proteins were purified by Ni‐NTA (Qiagen, Hilden, Germany), anion/cation chromatography, purified further by high‐resolution size exclusion chromatography and passed over Mustang E filters (Pall Life Sciences, Portsmouth, UK) to remove residual endotoxin from the preparations. The purity of the proteins was checked on a 12·5% sodium dodecyl sulphate‐polymerase gel electrophoresis (SDS‐PAGE) and the concentrations determined using optical density (OD)280 nm measurements and extinctions coefficients.

Non‐haemolytic (detoxified) Ply (dPly) was generated from native Ply and provided by Professor T. Mitchell, at the time of generation affiliated with the Laboratory of Infection and Immunity, Glasgow Biomedical Research Centre, University of Glasgow, Scotland (currently at University of Birmingham, UK) 28. As described previously for other Ply alleles 29, Ply from serotype 1 ST306 was cloned into pET33b (Merck, Kenilworth, NJ, USA), and purified on a nickel column.

### Cord blood mononuclear cell cultures

To measure *in‐vitro* T cell responses, CBMC were cultured for 96 h in duplicate in volumes of 250 µl at concentrations of 1 × 10^6^ cells/ml in RPMI‐1640/10% HI‐human AB serum (except PspA, for which cells were plated at a concentration of 2 × 10^6^ cells/ml) in 96 deep‐well microtitre plates in medium only (baseline control; unstimulated) or stimulated with dPly (1 µg/ml), PspA (a mix of families 1 and 2, 2·5 µg/ml each) and the mitogen phytohaemagglutinin (PHA) (1 µg/ml) as a positive control, at 37°C/5% CO_2_. Cells stimulated with PHA were cultured for 48 h only.

Innate immune responses to dPly were studied by culturing CBMC for 24 h at the same concentration of 1 × 10^6^ cells/ml in RPMI/10% non‐HI FCS in 96 deep‐well round‐bottomed microtitre plates (Nunc, Roskilde, Denmark) in medium alone or stimulated with dPly (1 µg/ml). Supernatants were stored at 4°C and cytokine responses were measured within 7 days.

### Cytokines

Levels of the cytokines IFN‐γ, IL‐5, IL‐13, IL‐6, IL‐10 and TNF‐α were measured using a time‐resolved fluorometry (TRF) method developed in‐house, as described elsewhere 30. In the TRF method, a lanthanide chelate prebound to a detection antibody is used to detect the cytokine and the measured fluorescence is read as the corresponding level of cytokine produced. Cytokine responses are presented as absolute levels (pg/ml) measured in culture supernatants, and as concentrations adjusted for baseline levels measured in CBMC cultured in medium only. In the absence of a detectable cytokine response, samples were assigned a level of 1·5 pg/ml.

### Nasopharyngeal swabs and bacteriology

Nasopharyngeal samples (NPS) were collected from participating PNG mothers at delivery and weekly from their infants during the first month of life. Standardized methodologies were used for collection, transportation and storage of the NPS and subsequent culturing, identification and isolation of *S. pneumoniae* 31, 32. Briefly, NPS were stored in 1 ml of skimmed milk–glucose–glycerol broth (SMGGB) at −70°C until further processing at the PNG Institute of Medical Research. After thawing and vortexing, 10 μl aliquots of the NPS in SMGGB were streaked onto horse blood agar, chocolate agar, gentamicin blood agar (5 μg/ml) and bacitracin chocolate agar (300 μg/ml). Plates were incubated overnight (18–24 h) at 37°C in a 5% CO_2_‐enriched atmosphere. Presumptive pneumococcal colonies were then cultured with an optochin disc and confirmed to be *S. pneumoniae* based on their susceptibility.

### Ethics

For both cohorts, written informed consent was obtained from the mothers and, when possible, their partners. Ethical approval was obtained from the Medical Research Advisory Committee of Papua New Guinea (MRAC 03/20) and the Princess Margaret Hospital (PMH) Ethics Committee in Perth, Australia (1083/EP; 768/EP; and 1942/EP).

### Statistical analysis

Non‐parametric Mann–Whitney *U*‐tests were performed to compare *in‐vitro* CBMC cytokine responses between the PNG and AUS cohorts, and within the PNG cohort to compare responses in relation to pneumococcal carriage in mother or child. Associations between relative CD4 T cell and APC CBMC proportions and *in‐vitro* CBMC cytokine responses were studied using multivariate linear regression analysis. Multivariate Cox regression analysis was performed to study the PNG within‐cohort associations between *in‐vitro* CBMC cytokine responses and the infants' age of acquisition of pneumococcal carriage within the first month of life, adjusting for confounding by maternal pneumococcal carriage at the time of birth and pneumococcal protein‐specific IgG antibody responses. All analyses were performed for both absolute and adjusted cytokine levels because it is unknown which of the two measures is most relevant biologically. All analyses were performed using spss version 15.0 (SPSS Inc., Chicago, IL, USA). GraphPad Prism Analytical Software was used to generate box‐and‐whisker plots for the cytokine response data (GraphPad, San Diego, CA, USA).

## Results

### Pneumococcal protein‐specific cytokine responses in PNG and AUS CBMC

**Figure 1:**
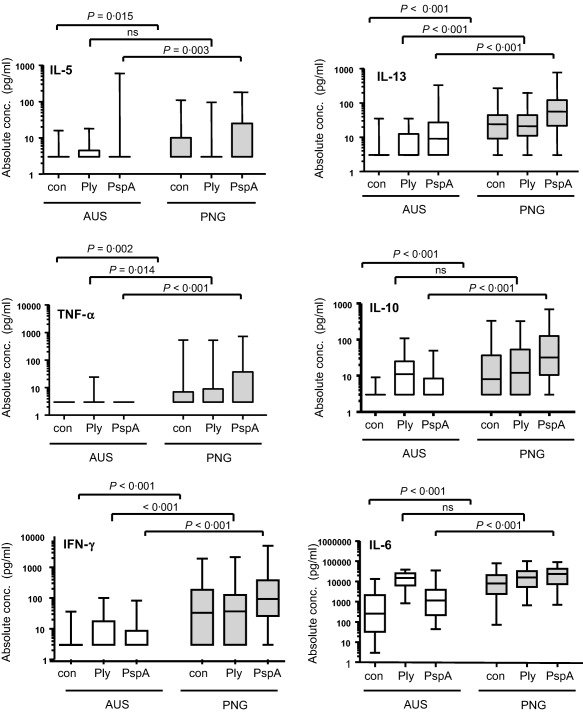
Absolute cytokine responses to pneumococcal surface protein A (PspA) and detoxified pneumolysin (dPly) in Papua New Guinean (PNG) and Australian (AUS) cord blood mononuclear cell (CBMC) cultures. CBMC of AUS (*n* = 33; white bars) and PNG (*n* = 84; grey bars) newborns were stimulated *in vitro* for 96 h with pneumococcal surface protein A (PspA), detoxified pneumolysin (dPly) or not stimulated. Absolute cytokine responses (pg/ml) measured in culture supernatants are presented as box‐and‐whisker plots.

Responses were low overall for all cytokines measured, except for IL‐6. CBMC of PNG infants were found to produce significantly higher baseline (non‐stimulated) levels of all measured cytokines (IL‐5, IL‐10, IL‐13, IFN‐γ, IL‐6 and TNF‐α) compared to CBMC of AUS infants (Fig. 1). In response to stimulation with PspA, PNG CBMC produced significantly higher levels of all measured cytokines compared to AUS CBMC cultures, when analysing absolute (Fig. 1) as well as adjusted cytokine levels (Table 1). In response to dPly, CBMC of PNG infants produced significantly higher absolute levels of IL‐13, IFN‐γ and TNF‐α than CBMC of AUS infants (Fig. 1), but these differences were no longer statistically significant when comparing adjusted cytokine levels (Table 1). CBMC of AUS newborns produced significantly higher IL‐6 levels in response to dPly stimulation compared to the PNG newborn cohort after adjusting for background levels (Table 1).

**Table 1:** Adjusted PspA, dPly and PHA cytokine responses in PNG and AUS CBMC cultures

	PNG CBMC (*n* = 84)	AUS CBMC (*n* = 33)	
	Median (IQR)	Median (IQR)	*P*‐value
I. PspA			
IFN‐γ	53·9 (1·5–177·6)	1·5 (1·5–6·4)	< 0·001
IL‐5	1·5 (1·5–17·5)	1·5 (1·5–1·5)	0·010
IL‐13	33·1 (10·6–75·3)	1·5 (1·5–24·2)	< 0·001
IL‐10	27·5 (6·3–89·2)	1·5 (1·5–5·3)	< 0·001
IL‐6	11 471 (3367–19 994)	481 (59–3369)	< 0·001
TNF‐α	1·5 (1·5–15·0)	1·5 (1·5–1·5)	< 0·001
II. dPly			
IFN‐γ	1·5 (1·5–30·8)	1·5 (1·5–11·3)	0·925
IL‐5	1·5 (1·5–1·5)	1·5 (1·5–1·7)	0·324
IL‐13	1·5 (1·5–10·3)	1·5 (1·5–9·6)	0·442
IL‐10	1·5 (1·5–12·3)	6·0 (1·5–22·1)	0·213
IL‐6	1972 (1·5–9203)	13 734 (5340–21 438)	< 0·001
TNF‐α	1·5 (1·5–1·5)	1·5 (1·5–1·5)	0·285
III. PHA			
IFN‐γ	135·8 (64·6–426·1)	28·4 (5·4–74·8)	0·019
IL‐5	1·5 (1·5–10·5)	1·5 (1·5–3·2)	0·307
IL‐13	51·2 (14·0–122·5)	35·5 (10·7–85·9)	< 0·001
IL‐10	25·5 (10·1–51·1)	22·1 (4·6–35·1)	0·117
IL‐6	16·1 (1·5–4476)	1113 (350–2300)	0·112
TNF‐α	1·5 (1·5–20·7)	1·5 (1·5–1·5)	0·003

Presented are median and interquartile ranges (IQR) of adjusted cytokine responses (pg/ml) to detoxified pneumolysin (dPly), pneumococcal surface protein A (PspA) or phytohaemagglutinin (PHA) (difference between cytokine levels in cord blood mononuclear cells (CBMC) cultures in stimulated minus unstimulated cultures) in Papua New Guinean (PNG) and Australian (AUS) newborns. IFN = interferon; IL = interleukin; TNF = tumour necrosis factor.

CBMC of both PNG and AUS newborns responded to stimulation with PHA, an APC‐dependent T cell mitogen, producing IFN‐γ as well as IL‐13, IL‐6 and measurable IL‐5 and IL‐10 responses, with adjusted IFN‐γ and IL‐5 responses being significantly higher in the PNG cohort (Table 1).

In summary, these findings show that CBMC of both PNG and AUS newborns are capable of producing Th1‐ and Th2‐associated cytokines as measured by PHA‐induced IFN‐γ, IL‐13 and other cytokine responses; that PspA‐specific CBMC responses are low in Australian newborns but significantly higher in PNG newborns; and that pneumolysin induces higher IL‐6 responses in AUS compared to PNG CBMC.

### Frequency of CD4 T cells present in PNG and AUS CBMC

We have reported previously that the relative proportion of CD4 T cells is higher in CBMC of PNG than of AUS newborns 33. We therefore explored whether the relative number of CD4 T cells present relates to the CBMC cytokine responses to PspA, dPly and PHA stimulation, as this supports that these responses would be produced by CD4 T cells. In line with earlier findings, the proportion of CD4‐high T cells was significantly higher in CBMC of PNG newborns [median = 38·3%, interquartile ranges (IQR) = 29·3–45·6] than in CBMC of AUS newborns (median = 30·2%, IQR = 26·7–35·3) (*P = *0·005), while the relative proportion of CD4‐low expressing APC was significantly lower in PNG CBMC (median = 14·3%, IQR = 10·2–18·9) than in AUS CBMC (median = 20·9%, IQR = 17·5–26·7) (*P* < 0·001). As summarized in Table 2, higher CBMC PspA‐IL‐5 and PspA‐IL‐13 responses were found to be associated significantly with a higher relative proportion of CD4 T cells. A similar significant association was found for PHA‐IL‐13. A negative significant association was found between CBMC PHA‐IFN‐γ responses and proportion of CD4‐low‐expressing APC. A positive significant association was found between the proportion of APC and dPly‐IL‐6 responses.

**Table 2:** Multivariate linear regression analysis studying independent associations between proportion of CD4‐T cell and APC in PNG and AUS CBMC in relation to CBMC *in‐vitro* PspA, dPly or PHA‐induced cytokine responses

	PspA		dPly		PHA	
	β ± s.e.	*P*‐value	β ± s.e.	*P*‐value	β ± s.e.	*P*‐value
IFN‐γ						
% CD4‐T cell	3·74 ± 6·20	0·548	−0·35 ± 0·61	0·569	−0·86 ± 9·65	0·930
% APC	−12·85 ± 8·48	0·134	0·41 ± 0·83	0·619	**−33·31 ± 13·37**	**0·013**
IL‐5						
% CD4‐T cell	**1·92 ± 0·91**	**0·039**	0·08 ± 0·13	0·541	0·22 ± 0·16	0·168
% APC	0·84 ± 1·25	0·505	−0·05 ± 0·18	0·780	−0·13 ± 0·21	0·539
IL‐13						
% CD4‐T cell	**3·08 ± 1·29**	**0·020**	0·18 ± 0·20	0·379	**3·17 ± 1·31**	**0·018**
% APC	2·05 ± 1·76	0·248	−0·15 ± 0·27	0·594	−0·88 ± 1·77	0·620
IL‐10						
% CD4‐T cell	0·64 ± 1·07	0·554	−0·10 ± 0·24	0·684	1·10 ± 0·97	0·258
% APC	0·25 ± 1·47	0·864	0·30 ± 0·32	0·355	0·38 ± 1·31	0·773
IL‐6						
% CD4‐T cell	98·71 ± 138·71	0·479	**−59·75 ± 131·56**	**0·651**	46·42 ± 119·09	0·698
% APC	13·47 ± 187·77	0·943	**650·06 ± 175·69**	**<0·001**	43·06 ± 161·20	0·790
TNF‐α						
% CD4‐T cell	0·47 ± 0·67	0·481	−0·003 ± 0·04	0·937	1·01 ± 1·92	0·603
% APC	1·30 ± 0·91	0·158	−0·01 ± 0·06	0·839	0·30 ± 2·60	0·908

Multivariate linear regression analysis was used to study associations between the number of CD4‐high T cells and CD4‐low antigen‐presenting cells (APC) as a proportion of cord blood mononuclear cells (CBMC) and background adjusted *in‐vitro* CBMC cytokine responses to pneumococcal surface protein A (PspA), detoxified pneumolysin (dPly), and phytohaemagglutinin (PHA) in Papua New Guinean (PNG) (*n* = 43) and Australian (AUS) (*n* = 33) newborns. IFN = interferon; IL = interleukin; TNF = tumour necrosis factor; s.e. = standard error.

These findings suggest that *in‐vitro* CBMC cytokine responses to PspA probably involve a CD4 T cell response, while responses to dPly are more likely to involve APC than CD4 T cells.

### Maternal pneumococcal carriage at the time of delivery and CBMC pneumococcal‐specific responses

Within the PNG cohort, associations between CBMC pneumococcal‐specific immune responses and active maternal pneumococcal carriage at the time of delivery were studied.

Twenty of the PNG study mothers carried pneumococci at the time of delivery and 42 were non‐carriers (for 22 mothers no carriage data were available). There were no statistically significant differences in cord blood cytokine responses to the pneumococcal proteins PspA and dPly between infants whose mothers carried pneumococci at the time of delivery and those who did not (Supporting information, Table S1).

### Cord pneumococcal‐specific cellular responses and risk for early pneumococcal colonization

Next we studied within the PNG cohort associations between CBMC pneumococcal‐specific immune responses and pneumococcal acquisition in the infants in the first month of life. NPS were collected from the infants at 1, 2, 3 and 4 weeks of age.

Of the 84 study infants, 48 were colonized within the first month of life and 21 were negative at each time‐point (for 15 infants, data were incomplete and censored at the last sequential time‐point). The cumulative pneumococcal acquisition rate in the PNG infants during the first month of life is shown in Supporting information, Fig. S1. Pneumococcal acquisition was comparable for children who received a dose of PCV at birth and children who did not (data not shown).

While CBMC pneumococcal‐specific cytokine responses were generally higher in infants who were not colonized within the first month of life compared to those who were, these differences did not reach statistical significance, except for (absolute) dPly‐IFN‐γ responses (Supporting information, Table S2). When adjusted cytokine levels were compared, no differences were found (data not shown).

Using multivariate Cox regression analysis and analysing adjusted cytokine levels, higher CBMC PspA‐IL‐5 and PspA‐IL‐6 responses were associated significantly with earlier pneumococcal colonization, while a significant protective effect was found for PspA‐IL‐10 and a similar trend for PspA‐IL‐13 (Table 3). No significant associations were found for dPly‐induced cytokine responses (Table 3) or PHA‐induced cytokine responses (Supporting information, Table S3).

**Table 3:** Cox regression analysis for associations between cord PspA and dPly‐specific cytokine responses and age of first pneumococcal colonization in the first month of life in PNG infants

	(a) Absolute cytokine levels		(b) Adjusted cytokine levels	
I. PspA	HR (95% CI)	*P*‐value	HR (95% CI)	*P*‐value
IFN‐γ	0·84 (0·46–1·52)	0·557	1·35 (0·85–2·15)	0·204
IL‐5	1·78 (0·595–5·31)	0·303	**2·67 (1·08–6·58)**	**0·033**
IL‐13	0·67 (0·24–1·83)	0·430	0·48 (0·22–1·02)	0·057
IL‐10	0·67 (0·21–2·14)	0·500	**0·40 (0·18–0·89)**	**0·025**
IL‐6	2·24 (0·41–12·12)	0·351	5·80 (1·66–20·20)	0·006
TNF‐α	1·18 (0·54–2·60)	0·680	1·05 (0·51–2·19)	0·889
II. dPly				
IFN‐γ	0·55 (0·27–1·09)	0·088	1·13 (0·57–2·25)	0·732
IL‐5	3·08 (0·92–10·28)	0·068	1·38 (0·28–6·75)	0·691
IL‐13	2·19 (0·56–8·51)	0·260	0·85 (0·29–2·46)	0·767
IL‐10	3·06 (0·93–10·08)	0·065	1·64 (0·69–3·92)	0·264
IL‐6	0·36 (0·09–1·36)	0·131	0·92 (0·69–1·24)	0·596
TNF‐α	0·69 (0·24–1·98)	0·492	0·26 (0·04–1·80)	0·172

Multivariate Cox regression models studying independent associations between cord blood mononuclear cells (CBMC) cytokine responses to pneumococcal surface protein A (PspA) (I) and detoxified pneumolysin (dPly) (II), and age of first upper respiratory tract pneumococcal colonization in the first 4 weeks of life in Papua New Guinean (PNG) infants, adjusted for maternal pneumococcal carriage at the time of delivery and cord plasma PspA‐families 1 and 2 (I), or Ply‐specific immunoglobulin (Ig)G antibody titres (II). Associations were studied for (a) absolute cytokine concentrations measured in culture supernatants and (b) concentrations adjusted for baseline levels present in non‐stimulated cultures. CI = confidence interval; HR = hazard ratio; IFN = interferon; IL = interleukin; TNF = tumour necrosis factor.

Because Ply is known to activate the innate immune system, we also assessed *in‐vitro* CBMC cytokine responses to dPly after 24 h of culture as a measure of innate immune responses, but no significant associations were found in relation to age of first colonization (Supporting information, Table S4).

## Discussion

Pneumococcal colonization of the upper respiratory tract is an immediate precursor to pneumococcal disease. In settings with a high burden of pneumococcal disease such as PNG, infants become colonized rapidly and disease is likely to follow. For example, in PNG 50% of infants are colonized by 17–18 days of age 5. Moreover, in high‐risk settings pneumococcal colonization is intense, persistent and continues into adulthood. A possible explanation for this early and persistent carriage might be a distortion in the development of pneumococcal protective immune responses early in life. We show here that in infants born in a highly endemic setting such as the highlands of PNG, pneumococcal‐specific cellular immune responses at birth differ from those found in infants born in a low endemic setting in Australia, and are associated with higher risk for early pneumococcal colonization.

Compared to Australian newborns, CBMC responses to the pneumococcal protein PspA but not dPly were significantly higher. We postulate that these PspA responses are CD4 T cell‐mediated based on the observation that PspA‐induced IL‐5 and IL‐13 responses, cytokines produced by Th2 cells, were associated significantly with the proportion of cord CD4 T cells present in CBMC samples. In contrast to PspA, we believe that dPly predominantly stimulates cord innate immune cells. Pneumolysin has been described to activate innate immune cells by signalling through the NLRP3 inflammasome complex 34, resulting in a proinflammatory response that may attract pneumococcal*‐*specific T cells 35, and induce the development and activation of protective Th17 responses, in particular in the nasopharynx 36. While a few studies have reported on pneumolysin‐specific Th1 and Th17 responses in peripheral blood, these responses seem less apparent than responses in nasopharynx‐associated lymphoid tissue 21, 37. We postulate that, particularly in the neonatal period, the innate immune signalling properties ascribed to pneumolysin may predominate over that of T cell responses. We have reported previously that AUS CBMC produce higher cytokine responses to innate immune signals than PNG CBMC 26 which, as we showed in a subsequent study, is explained by the higher proportion as well as functionally more responsive status of APC in AUS compared to PNG CBMC 33, 38. We also found cord blood CD4 T cell cytokine responses to be similar in PNG and AUS CBMC when cultured in the presence of adult APC and at the same APC : T cell ratio; however, when comparing *in‐vitro* CBMC cultures as reported here, T cell cytokine responses may differ due to variation in the APC : T cell ratio, APC function, as well as possible earlier antigen experience of the neonatal immune system. Altogether, these observations support our suggestion that the elevated PspA response in PNG newborns is probably CD4 T cell‐mediated and the higher dPly response in AUS newborns is APC‐derived; however, we acknowledge that in this study we have not performed any experiments that directly identify the cell sources and their relative contribution to the PspA‐ and dPly‐induced cytokine responses.

We suggest further that the observed elevated PspA response in PNG newborns is, at least to an extent, antigen‐specific and not simply the result of an overall heightened cytokine response of PNG cord T cells. In preliminary experiments not reported here, we stimulated CBMC with a hypothetical protein of a deep‐sea hyperthermophilic, anaerobic archaeon, *Pyrococcus horikoshii* (European Nucleotide Archive Accession number: BAA29275), to which human newborns are not expected to have been exposed, and found that neither PNG nor AUS CBMC responded to this foreign protein, hence supporting that the PNG CBMC do not respond to every protein. Further indirect support for our hypothesis that the elevated cord PspA‐induced immune responses are specific is the finding of their significant association with early onset of pneumococcal colonization in PNG infants, independent of maternally derived pneumococcal protein antibodies, while such associations were not found for dPly‐ and PHA‐induced responses.

We found that cord blood PspA‐IL‐10 responses were associated with a protective effect by delaying the onset of colonization, while elevated cord blood PspA‐IL‐5 and PspA‐IL‐6 responses were associated with an increased risk of earlier colonization. A similar inverse association between cord blood T cell IL‐10 and IL‐5 responses and risk for respiratory infections has been described previously in a longitudinal birth cohort following children for 5 years: in this cohort, children with a low IL‐10/high IL‐5 T cell response phenotype at birth were at a significantly higher risk of all grades of acute respiratory infection relative to children with the more resistant high IL‐10/low IL‐5 phenotype 39. A recent study from Copenhagen showed that elevated *in‐vitro* IL‐5 and TNF‐α responses to *Haemophilus influenzae*, *Moraxella catarrhalis* and *S. pneumoniae* measured in 6‐month‐old children were predictive of an increased incidence of respiratory infections during the first 3 years of life 40. Although it is not clear how enhanced proinflammatory T cell responses at birth might increase the subsequent risk of pneumococcal colonization, as we observed here, our findings suggest a role for an immunological pathway that is activated *in utero* that limits the clearance of *S. pneumoniae* colonizing the upper respiratory tract in infants in high‐risk areas such as PNG.

Similar phenomena have been shown for helminths and malarial parasitic infections in pregnancy, where pathogen‐specific T cell responses induced *in utero* have long‐term negative consequences for the infants' susceptibility and development of immune responses to these parasites 41, 42, 43, 44, 45, 46, 47, 48, 49. Accordingly, *in‐utero* infection with cytomegalovirus (CMV) has been found to result in *in‐utero* activation of fetal CMV T cell responses that, compared to adult responses, are ‘functionally exhausted’, with a limited capacity to control CMV replication 50, 51. Although pneumococcal‐specific cord blood cellular cytokine responses were lower overall in infants born to mothers who were pneumococcal carriers at the time of delivery, we were not able to find significant differences compared with responses of infants born to mothers who were non‐carriers: we believe that a more extensive upper respiratory tract pneumococcal carriage study during pregnancy would be needed, including a larger population size to estimate more clearly the effect of maternal carriage on immune priming. Therefore, we can only speculate that the elevated PspA response in PNG newborns is the result of a similar phenomenon of *in‐utero* activation of fetal immune responses occurring in highly endemic areas where adults, including pregnant women, are exposed continuously to pneumococci 5, 52.

We acknowledge that sample numbers in our study were relatively low, and that the study had not been designed to assess maternal pneumococcal exposure accurately during pregnancy. Further studies are therefore needed to understand the mechanisms of how *in‐utero* fetal pneumococcal immune responses are activated. Despite the widespread use of *in‐vitro* or *ex‐vivo* cell culture systems to investigate *in‐vivo* human immune responses, there is no consensus on the use of absolute cytokine levels or that of baseline‐adjusted responses. Considering the high baseline cytokine levels that we observed in our PNG cell cultures, we chose to analyse and present both parameters. As the analyses were not adjusted for multiple testing, it is possible that the statistical significance of results may be due to chance.

In summary, we have demonstrated that in an area highly endemic for *S. pneumoniae* infections, infants may display elevated cellular immune responses to the pneumococcal protein PspA at birth, due possibly to a higher frequency of ‘sensitized’ (specific) T cells that may contribute to the risk of early pneumococcal colonization. This may explain the high risk for early and persistent pneumococcal colonization in high‐risk populations, and warrants studies into the appropriate adjuvanting of pneumococcal protein vaccines currently in development in order to induce the appropriate effector responses, in particular when neonatal immunization strategies are to be considered 11, 53.

## Author contributions

D. L., P. R., P. H. and W. P. and conceived and designed the PNG neonatal pneumococcal conjugate vaccination trial. A. V. D. B., P. R. and P. H. conceived and designed the immunological experiments. S. P. conceived and conducted the Australian cohort trial. J. F., M. N. S. and C. E. D. conducted the immunological experiments. A. M. supervised the bacteriology. J. F., P. R., D. S. and A. V. D. B. conducted the data analysis and interpretation. J. F. wrote the first draft of the manuscript and final version with support from A. V. D. B.; A. V. D. B., D. S., P. R., D. L., W. P., S. P. and P. H. critically reviewed and edited the final version. All authors approved the final version of the manuscript.

## Disclosure

P. R. has been a member of vaccine advisory boards for Wyeth and CSL Ltd and has received institutional funding for investigator‐initiated research from GlaxoSmithKline (GSK) Biologicals and Merck and received travel support from Pfizer and Baxter to present study data at international meetings. D. L. has been a member of the GSK Australia Pneumococcal–*Haemophilus influenzae–*Protein D conjugate vaccine (‘Synflorix’) Advisory Panel, has received support from Pfizer Australia and GSK Australia to attend conferences, has received an honorarium from Merck Vaccines to give a seminar at their offices in Pennsylvania and to attend a conference and is an investigator on an investigator‐initiated research grant funded by Pfizer Australia. W. P. has received travel scholarship from Pfizer™ to attend ISPPD8 in Iguacu Falls, Brazil and a co‐investigator in a Pfizer Investigator Initiated Grant. A. V. D. B. has received support from Pfizer Australia and GSK Australia to attend conferences and received a Pfizer‐supported Robert Austrian Award in Pneumococcal Vaccinology, and was previously an employee of Crucell/Janssen Pharmaceuticals, Johnson and Johnson. All other authors declare no disclosures.

## Supporting information

Additional supporting information may be found in the online version of this article at the publisher's web‐site.

**Fig. S1**. Cumulative pneumococcal acquisition rate in Papua New Guinean infants in the first year of life.

**Table S1**. CBMC PspA and dPly cytokine responses for PNG infants born to mothers who were carriers or non‐carriers of pneumococci at the time of delivery.
**Table S2**. PspA and dPly cytokine responses in CBMC cultures of PNG infants in relation to colonization in the first month of life.
**Table S3**. Cox regression analysis for associations between cord PHA‐specific cytokine responses and age of first pneumococcal colonization in the 1st month of life in PNG infants.
**Table S4**. Cox regression analysis for associations between CBMC innate dPly‐induced cytokine responses and age of pneumococcal colonization in 1st month of life in PNG study infants.

## References

[1] O'Brien KL , Wolfson LJ , Watt JP *et al* Burden of disease caused by *Streptococcus pneumoniae* in children younger than 5 years: global estimates. Lancet 2009; 374:893–902. [TQ1] 19748398 10.1016/S0140-6736(09)61204-6

[2] Whitney CG , Goldblatt D , O'Brien KL. Dosing schedules for pneumococcal conjugate vaccine: considerations for policy makers. Pediatr Infect Dis J 2014; 33 (Suppl 2):S172–81. 24336059 10.1097/INF.0000000000000076 PMC3940379

[3] Bogaert D , De Groot R , Hermans PW. *Streptococcus pneumoniae* colonisation: the key to pneumococcal disease. Lancet Infect Dis 2004; 4: 144–54. 14998500 10.1016/S1473-3099(04)00938-7

[4] Simell B , Auranen K , Kayhty H *et al* The fundamental link between pneumococcal carriage and disease. Expert Rev Vaccines 2012; 11:841–55. 22913260 10.1586/erv.12.53

[5] Francis JP , Richmond PC , Pomat WS *et al* Maternal antibodies to pneumolysin but not to pneumococcal surface protein A delay early pneumococcal carriage in high‐risk Papua New Guinean infants. Clin Vaccine Immunol 2009; 16:1633–8. 19776196 10.1128/CVI.00247-09 PMC2772384

[6] Bacterial etiology of serious infections in young infants in developing countries: results of a multicenter study. The WHO Young Infants Study Group. Pediatr Infect Dis J 1999; 18:S17–22. 10530569 10.1097/00006454-199910001-00004

[7] Duke T. Neonatal pneumonia in developing countries. Arch Dis Child Fetal Neonatal Edn 2005; 90:F211–9. 10.1136/adc.2003.048108 PMC1721897 15846010

[8] Torzillo PJ , Hanna JN , Morey F , Gratten M , Dixon J , Erlich J. Invasive pneumococcal disease in central Australia. Med J Aust 1995; 162:182–6. 7877538 10.5694/j.1326-5377.1995.tb126016a.x

[9] Waters D , Jawad I , Ahmad A *et al* Aetiology of community‐acquired neonatal sepsis in low and middle income countries. J Glob Health 2011; 1:154–70. 23198116 PMC3484773

[10] Kothari N , Genschmer KR , Kothari S *et al* Preparation and testing of a Vi conjugate vaccine using pneumococcal surface protein A (PspA) from *Streptococcus pneumoniae* as the carrier protein. Vaccine 2014; 32:5755–60. 25171842 10.1016/j.vaccine.2014.08.041

[11] Kothari N , Kothari S , Choi YJ *et al* A bivalent conjugate vaccine containing PspA families 1 and 2 has the potential to protect against a wide range of *Streptococcus pneumoniae* strains and *Salmonella Typhi* . Vaccine 2015; 33:783–8. 25545593 10.1016/j.vaccine.2014.12.032

[12] Linder A , Hollingshead S , Janulczyk R , Christensson B , Akesson P. Human antibody response towards the pneumococcal surface proteins PspA and PspC during invasive pneumococcal infection. Vaccine 2007; 25:341–5. 16996173 10.1016/j.vaccine.2006.07.028

[13] Marriott HM , Mitchell TJ , Dockrell DH. Pneumolysin: a double‐edged sword during the host‐pathogen interaction. Curr Mol Med 2008; 8:497–509. 18781957 10.2174/156652408785747924

[14] Darrieux M , Miyaji EN , Ferreira DM *et al* Fusion proteins containing family 1 and family 2 PspA fragments elicit protection against *Streptococcus pneumoniae* that correlates with antibody‐mediated enhancement of complement deposition. Infect Immun 2007; 75:5930–8. 17923518 10.1128/IAI.00940-07 PMC2168346

[15] Ochs MM , Bartlett W , Briles DE *et al* Vaccine‐induced human antibodies to PspA augment complement C3 deposition on *Streptococcus pneumoniae* . Microb Pathog 2008; 44:204–14. 18006268 10.1016/j.micpath.2007.09.007 PMC2288783

[16] Jedrzejas MJ. Unveiling molecular mechanisms of bacterial surface proteins: *Streptococcus pneumoniae* as a model organism for structural studies. Cell Mol Life Sci 2007; 64:2799–822. 17687514 10.1007/s00018-007-7125-8 PMC11136149

[17] Miyaji EN , Dias WO , Tanizaki MM , Leite LC. Protective efficacy of PspA (pneumococcal surface protein A)‐based DNA vaccines: contribution of both humoral and cellular immune responses. FEMS Immunol Med Microbiol 2003; 37:53–7. 12770760 10.1016/S0928-8244(03)00108-1

[18] Palaniappan R , Singh S , Singh UP *et al* Differential PsaA‐, PspA‐, PspC‐, and PdB‐specific immune responses in a mouse model of pneumococcal carriage. Infect Immun 2005; 73:1006–13. 15664944 10.1128/IAI.73.2.1006-1013.2005 PMC547096

[19] Francis JP , Richmond PC , Michael A *et al* A longitudinal study of natural antibody development to pneumococcal surface protein A families 1 and 2 in Papua New Guinean Highland children. Pneumonia 2016; 8:12. 10.1186/s41479-016-0014-x PMC5471893 28702291

[20] Sharma SK , Roumanes D , Almudevar A , Mosmann TR , Pichichero ME. CD4+ T‐cell responses among adults and young children in response to *Streptococcus pneumoniae* and *Haemophilus influenzae* vaccine candidate protein antigens. Vaccine 2013; 31:3090–7. 23632305 10.1016/j.vaccine.2013.03.060 PMC3777711

[21] Zhang Q , Bagrade L , Bernatoniene J *et al* Low CD4 T cell immunity to pneumolysin is associated with nasopharyngeal carriage of pneumococci in children. J Infect Dis 2007; 195:1194–202. 17357058 10.1086/512617

[22] van den Biggelaar AH , Pomat WS , Phuanukoonnon S *et al* Effect of early carriage of *Streptococcus pneumoniae* on the development of pneumococcal protein‐specific cellular immune responses in infancy. Pediatr Infect Dis J 2012; 31:243–8. 22189528 10.1097/INF.0b013e318245a5a8

[23] Holt PG , Jones CA. The development of the immune system during pregnancy and early life. Allergy 2000; 55:688–97. 10955693 10.1034/j.1398-9995.2000.00118.x

[24] Gratten M , Gratten H , Poli A , Carrad E , Raymer M , Koki G. Colonisation of *Haemophilus influenzae* and S*treptococcus pneumoniae* in the upper respiratory tract of neonates in Papua New Guinea: primary acquisition, duration of carriage, and relationship to carriage in mothers. Biol Neonate 1986; 50:114–20. 3489488 10.1159/000242576

[25] Watson K , Carville K , Bowman J *et al* Upper respiratory tract bacterial carriage in Aboriginal and non‐Aboriginal children in a semi‐arid area of Western Australia. Pediatr Infect Dis J 2006; 25:782–90. 16940834 10.1097/01.inf.0000232705.49634.68

[26] van den Biggelaar AH , Prescott SL , Roponen M *et al* Neonatal innate cytokine responses to BCG controlling T‐cell development vary between populations. J Allergy Clin Immunol 2009; 124:544–50, 50 e1–2. 19500827 10.1016/j.jaci.2009.03.040

[27] Phuanukoonnon S , Reeder JC , Pomat WS *et al* A neonatal pneumococcal conjugate vaccine trial in Papua New Guinea: study population, methods and operational challenges. PNG Med J 2010; 53:191–206. 23163191

[28] Kirkham LA , Kerr AR , Douce GR *et al* Construction and immunological characterization of a novel nontoxic protective pneumolysin mutant for use in future pneumococcal vaccines. Infect Immun 2006; 74:586–93. 16369015 10.1128/IAI.74.1.586-593.2006 PMC1346677

[29] Kirkham LA , Jefferies JM , Kerr AR *et al* Identification of invasive serotype 1 pneumococcal isolates that express nonhemolytic pneumolysin. J Clin Microbiol 2006; 44:151–9. 16390963 10.1128/JCM.44.1.151-159.2006 PMC1351962

[30] Sharp MJ , Rowe J , Kusel M , Sly PD , Holt PG. Specific patterns of responsiveness to microbial antigens staphylococcal enterotoxin B and purified protein derivative by cord blood mononuclear cells are predictive of risk for development of atopic dermatitis. Clin Exp Allergy 2003; 33:435–41. 12680857 10.1046/j.1365-2222.2003.01627.x

[31] Aho C , Michael A , Yoannes M *et al* Limited impact of neonatal or early infant schedules of 7‐valent pneumooccal conjugate vaccination on nasopharyngeal carriage of *Streptococcus pneumoniae* in Papua New Guinean children: a randomized controlled trial. Vaccine Rep 2016; 6:36–43. 10.1016/j.vacrep.2016.08.002 PMC5446595 28580433

[32] Satzke C , Turner P , Virolainen‐Julkunen A *et al* Standard method for detecting upper respiratory carriage of *Streptococcus pneumoniae*: updated recommendations from the World Health Organization Pneumococcal Carriage Working Group. Vaccine 2013; 32:165–79. 24331112 10.1016/j.vaccine.2013.08.062

[33] Lisciandro JG , Prescott SL , Nadal‐Sims MA *et al* Neonatal antigen presenting cells are functionally more quiescent in children born under traditional compared to modern environmental conditions. J Allergy Clin Immunol 2012; 130:1167–74. 22818765 10.1016/j.jaci.2012.06.005

[34] McNeela EA , Burke A , Neill DR *et al* Pneumolysin activates the NLRP3 inflammasome and promotes proinflammatory cytokines independently of TLR4. PLOS Pathog 2010; 6:e1001191. 21085613 10.1371/journal.ppat.1001191 PMC2978728

[35] Kadioglu A , Coward W , Colston MJ , Hewitt CR , Andrew PW. CD4‐T‐lymphocyte interactions with pneumolysin and pneumococci suggest a crucial protective role in the host response to pneumococcal infection. Infect Immun 2004; 72:2689–97. 10.1128/IAI.72.5.2689-2697.2004 PMC387852 15102777

[36] Gray C , Ahmed MS , Mubarak A *et al* Activation of memory Th17 cells by domain 4 pneumolysin in human nasopharynx‐associated lymphoid tissue and its association with pneumococcal carriage. Mucosal Immunol 2014; 7:705–17. 24220296 10.1038/mi.2013.89

[37] Mureithi MW , Finn A , Ota MO *et al* T cell memory response to pneumococcal protein antigens in an area of high pneumococcal carriage and disease. J Infect Dis 2009; 200:783–93. 19642930 10.1086/605023

[38] Lisciandro JG , Prescott SL , Nadal‐Sims MG *et al* Comparison of neonatal T regulatory cell function in Papua New Guinean and Australian newborns. Pediatr Allergy Immunol 2011; 23:173–80. 22192086 10.1111/j.1399-3038.2011.01242.x

[39] Zhang G , Rowe J , Kusel M *et al* Interleukin‐10/interleukin‐5 responses at birth predict risk for respiratory infections in children with atopic family history. Am J Respir Crit Care Med 2009; 179:205–11. 18996999 10.1164/rccm.200803-438OC

[40] Vissing NH , Larsen JM , Rasmussen MA *et al* Susceptibility to lower respiratory infections in childhood is associated with perturbation of the cytokine response to pathogenic airway bacteria. Pediatr Infect Dis J 2016; 35:561–6. 26910587 10.1097/INF.0000000000001092

[41] Malhotra I , Ouma JH , Wamachi A *et al* Influence of maternal filariasis on childhood infection and immunity to *Wuchereria bancrofti* in Kenya. Infect Immun 2003; 71:5231–7. 12933869 10.1128/IAI.71.9.5231-5237.2003 PMC187356

[42] Malhotra I , Dent A , Mungai P *et al* Can prenatal malaria exposure produce an immune tolerant phenotype? A prospective birth cohort study in Kenya. PLOS Med 2009; 6:e1000116. 19636353 10.1371/journal.pmed.1000116 PMC2707618

[43] Elson LH , Days A , Calvopina M *et al* *In utero* exposure to *Onchocerca volvulus*: relationship to subsequent infection intensity and cellular immune responsiveness. Infect Immun 1996; 64:5061–5. 8945547 10.1128/iai.64.12.5061-5065.1996 PMC174489

[44] Mehta RS , Rodriguez A , Chico M *et al* Maternal geohelminth infections are associated with an increased susceptibility to geohelminth infection in children: a case–control study. PLOS Negl Trop Dis 2012; 6:e1753. 22848773 10.1371/journal.pntd.0001753 PMC3404107

[45] Mpairwe H , Tweyongyere R , Elliott A. Pregnancy and helminth infections. Parasite Immunol 2014; 36:328–37. 24471654 10.1111/pim.12101 PMC4260141

[46] Malhotra I , Mungai P , Muchiri E *et al* Distinct Th1‐ and Th2‐type prenatal cytokine responses to *Plasmodium falciparum* erythrocyte invasion ligands. Infect Immun 2005; 73:3462–70. 15908375 10.1128/IAI.73.6.3462-3470.2005 PMC1111871

[47] Broen K , Brustoski K , Engelmann I , Luty AJ. Placental *Plasmodium falciparum* infection: causes and consequences of in utero sensitization to parasite antigens. Mol Biochem Parasitol 2007; 151:1–8. 17081634 10.1016/j.molbiopara.2006.10.001

[48] Brustoski K , Moller U , Kramer M *et al* IFN‐gamma and IL‐10 mediate parasite‐specific immune responses of cord blood cells induced by pregnancy‐associated *Plasmodium falciparum* malaria. J Immunol 2005; 174:1738–45. 15661939 10.4049/jimmunol.174.3.1738

[49] King CL , Malhotra I , Wamachi A *et al* Acquired immune responses to *Plasmodium falciparum* merozoite surface protein‐1 in the human fetus. J Immunol 2002; 168:356–64. 11751981 10.4049/jimmunol.168.1.356

[50] Vermijlen D , Brouwer M , Donner C *et al* Human cytomegalovirus elicits fetal gammadelta T cell responses *in utero* . J Exp Med 2010; 207:807–21. 20368575 10.1084/jem.20090348 PMC2856038

[51] Huygens A , Lecomte S , Tackoen M *et al* Functional exhaustion limits CD4+ and CD8+ T‐Cell responses to congenital cytomegalovirus infection. J Infect Dis 2015; 212:484–94. 25657256 10.1093/infdis/jiv071

[52] Conklin LM , Bigogo G , Jagero G *et al* High *Streptococcus pneumoniae* colonization prevalence among HIV‐infected Kenyan parents in the year before pneumococcal conjugate vaccine introduction. BMC Infect Dis 2016; 16:18. 26774803 10.1186/s12879-015-1312-2 PMC4715316

[53] Oliveira ML , Miyaji EN , Ferreira DM *et al* Combination of pneumococcal surface protein A (PspA) with whole cell pertussis vaccine increases protection against pneumococcal challenge in mice. PLOS ONE 2010; 5:e10863. 20523738 10.1371/journal.pone.0010863 PMC2877721

