# Supplementary figures and images for: Cord blood Streptococcus pneumoniae‐specific cellular immune responses predict early pneumococcal carriage in high‐risk infants in Papua New Guinea
Source: Clin Exp Immunol. 2016 Dec 18;187(3):408–17. doi: 10.1111/cei.12902 (PMC5290304; doi:10.1111/cei.12902)

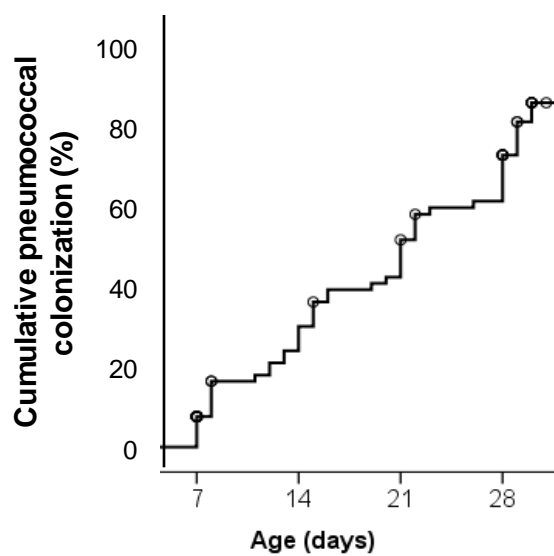

Supplementary Figure 1

Supplement: Supplementary file 1 — Fig. S1. Cumulative pneumococcal acquisition rate in Papua New Guinean infants in the first year of life. [file CEI-187-408-s001.pdf]
