# Supplementary material for: Cord blood Streptococcus pneumoniae‐specific cellular immune responses predict early pneumococcal carriage in high‐risk infants in Papua New Guinea
Source: Clin Exp Immunol. 2016 Dec 18;187(3):408–17. doi: 10.1111/cei.12902 (PMC5290304; doi:10.1111/cei.12902)
Supplement: Supplementary file 2 — Table S1. CBMC PspA and dPly cytokine responses for PNG infants born to mothers who were carriers or non‐carriers of pneumococci at the time of delivery. Table S2. PspA and dPly cytokine responses in CBMC cultures of PNG infants in relation to colonization in the first month of life. Table S3. Cox regression analysis for associations between cord PHA‐specific cytokine responses and age of first pneumococcal colonization in the 1st month of life in PNG infants. Table S4. Cox regression analysis for associations between CBMC innate dPly‐induced cytokine responses and age of pneumococcal colonization in 1st month of life in PNG study infants. [file CEI-187-408-s002.doc]

**Online Supplementary Table 1: CBMC PspA and dPly cytokine responses for PNG infants born to mothers who were carriers or non-carriers of pneumococci at the time of delivery**

|  | **Absolute levels** | |  | **Adjusted levels** | |  |
| --- | --- | --- | --- | --- | --- | --- |
|  | **Mother, non-carrier** | **Mother, carrier** |  | **Mother, non-carrier** | **Mother, carrier** |  |
|  | Median (IQR) | Median (IQR) | P-value | Median (IQR) | Median (IQR) | P-value |
| IFNγ-con | 23.5 (3.0-192.2) | 28.3 (1.5 – 134.9) | 0.687 | ----- | ----- | ----- |
| IFNγ-PspA | 83.0 (3.0 – 400.8) | 57.3 (38.8 – 207.1) | 0.918 | 45.8 (1.5 – 252.7) | 38.6 (1.8 – 168.9) | 0.769 |
| IFNγ-Ply | 31.7 (1.5 – 133.3) | 27.5 (1.5 – 100.3) | 0.635 | 1.5 (1.5 – 28.9) | 1.5 (1.5 – 1.5) | 0.058 |
| IL5-con | 1.5 (1.5 – 133.3) | 1.5 (1.5 – 14.5) | 0.593 | ----- | ----- | ----- |
| IL5-PspA | 1.5 (1.5 – 22.7) | 1.5 (1.5 – 20.3) | 0.420 | 1.5 (1.5 – 14.2) | 1.5 (1.5 – 17.2) | 0.561 |
| IL5-Ply | 1.5 (1.5 – 1.5) | 1.5 (1.5 – 1.5) | 0.891 | 1.5 (1.5 – 1.5) | 1.5 (1.5 – 1.5) | 0.514 |
| IL13-con | 19.2 (6.8 – 50.8) | 23.4 (8.1 – 39.0) | 0.844 | ----- | ----- | ----- |
| IL13-PspA | 46.7 (17.7 – 139.4) | 55.1 (27.5 – 94.6) | 0.616 | 26.6 (3.7 – 86.4) | 25.0 (11.0 – 65.1) | 0.888 |
| IL13-Ply | 14.1 (17.7- 139.4) | 20.4 (11.8 – 35.5) | 0.281 | 1.5 (1.5 – 5.2) | 1.5 (1.5 – 10.1) | 0.891 |
| IL10-con | 1.5 (1.5 – 28.1) | 9.3 (1.5 – 69.7) | 0.314 | ----- | ----- | ----- |
| IL10-PspA | 31.2 (9.8 – 104.7) | 24.0 (8.5 – 95.2) | 0.937 | 28.2 (6.8 – 57.6) | 15.5 (3.7 – 64.5) | 0.648 |
| IL10-Ply | 11.2 (1.5 – 44.2) | 8.0 (1.5 – 57.9) | 0.912 | 3.0 (1.5 – 10.2) | 1.5 (1.5 – 9.1) | 0.564 |
| IL6-con | 6579 (2329 – 18386) | 8118 (3298 – 22706) | 0.451 | ----- | ----- | ----- |
| IL6-PspA | 23512 (5898 - 42420) | 22055 (8610 – 41533) | 0.838 | 11271 (3188 – 17382) | 9695 (2489 – 18174) | 0.730 |
| IL6-Ply | 12386 (4192 – 32576) | 18144 (7024 – 28350) | 0.683 | 2127 (1.5 – 10635) | 944 (1.5 – 10513) | 0.328 |
| TNFα-con | 1.5 (1.5 - 1.5) | 1.5 (1.5 – 18.6) | 0.561 | ----- | ----- | ----- |
| TNFα-PspA | 1.5 (1.5 – 27.2) | 1.5 (1.5 – 33.4) | 0.479 | 1.5 (1.5 – 17.0) | 1.5 (1.5 – 1.5) | 0.165 |
| TNFα-Ply | 1.5 (1.5 – 10.6) | 1.5 (1.5 – 1.5) | 0.769 | 1.5 (1.5 – 1.5) | 1.5 (1.5 – 1.5) | 0.988 |

CBMC of PNG newborns were stimulated with the pneumococcal proteins PspA and dPly or not stimulated (controls; con). Absolute cytokine levels (pg/ml) as measured in culture supernatants as well as adjusted cytokine levels (levels in stimulated cultures minus levels in non-stimulated control cultures) were compared for newborns whose mothers carried (n = 20) or did not carry (n = 42) pneumococci at the time of delivery.

**Online Supplementary** **Table 2: PspA and dPly cytokine responses in CBMC cultures of PNG infants in relation to colonization in the first month of life**

|  | **Infant, Non-carrier (n = 21)** | **Infant, Carrier (n = 48)** |  |
| --- | --- | --- | --- |
|  | Median (IQR) | Median (IQR) | P-value |
| **IFN-γ**  Control  PspA  dPly | 44.3 (1.5 – 196.4)  113.7 (49.0 – 268.7)  102.3 (22.9 – 202.5) | 15.1 (1.5 – 124.7)  47.6 (7.0 – 240.5)  21.2 (1.5 – 65.7) | 0.442  0.237  0.046 |
| **IL-5**  Control  PspA  dPly | 4.6 (1.5 – 17.5)  11.7 (1.5 – 32.1)  1.5 (1.5 – 16.5) | 1.5 (1.5 – 10.5)  1.5 (1.5 – 25.6)  1.5 (1.5 – 1.5) | 0.346  0.227  0.331 |
| **IL-13**  Control  PspA  dPly | 26.4 (12.1 – 63.8)  97.0 (32.0 – 148.7)  32.20 (9.5 – 73.9) | 18.2 (6.9 – 44.7)  55.1 (18.4 – 128.7)  18.1 (10.2 – 33.6) | 0.238  0.321  0.326 |
| **IL-10**  Control  PspA  dPly | 5.4 (1.5 – 40.6)  13.6 (1.5 – 13.0.3)  15.1 (1.5 – 78.8) | 6.0 (1.5 – 31.1)  31.2 (8.5 – 95.2)  11.2 (1.5 – 46.7) | 0.869  0.575  0.648 |
| **IL-6**  Control  PspA  dPly | 4713 (1537 – 34622)  18294 (3708 – 47199)  13664 (3383 – 36702) | 6545 (2545 – 17499)  15805 (10157 – 35581)  15277 (5248 – 25526) | 0.877  0.775  0.848 |
| **TNF-α**  Control  PspA  dPly | 1.5 (1.5 – 8.0)  1.5 (1.5 – 37.1)  1.5 (1.5 – 12.3) | 1.5 (1.5 – 1.5)  1.5 (1.5 – 32.3)  1.5 (1.5 – 1.5) | 1.000  0.950  0.753 |

CBMC of PNG newborns were stimulated with the pneumococcal proteins pneumolysin (dPly) and pneumococcal surface protein A (PspA) or not stimulated (controls). Absolute cytokine levels (pg/ml) were measured in culture supernatants, and the groups’ median and interquartile ranges (IQR) are presented for newborns carrying (n = 48) or, not carrying (n = 21) pneumococci in their upper respiratory tract within 1 month of life.

**Online Supplementary Table 3. Cox regression analysis for associations between cord PHA-specific cytokine responses and age of first pneumococcal colonization in the 1^st^** month of life in PNG infants

|  | **Model I** | | **Model II** | |
| --- | --- | --- | --- | --- |
|  | HR (95% CI) | P-value | HR (95% CI) | P-value |
| PHA-IFNγ | 1.00 (0.63-1.61) | 0.987 | 1.55 (0.79-3.03) | 0.198 |
| PHA-IL5 | 0.70 (0.27-1.81) | 0.557 | 0.36 (0.11-1.21) | 0.098 |
| PHA-IL13 | 0.75 (0.39-1.41) | 0.367 | 1.02 (0.49-2.11) | 0.969 |
| PHA-IL10 | 1.06 (0.58-1.92) | 0.852 | 1.35 (0.68-2.68) | 0.389 |
| PHA-IL6 | 1.12 (0.90-1.40) | 0.317 | 1.11 (0.87-1.42) | 0.396 |
| PHA-TNFα | 1.12 (0.63-1.99) | 0.707 | 0.80 (0.37-1.72) | 0.570 |

Multivariate Cox regression models studying independent associations between CBMC cytokine responses to PHA (adjusted levels) and age of first upper respiratory tract pneumococcal colonization in the first 4 weeks of life in PNG infants, adjusted for maternal pneumococcal carriage at the time of delivery (model I), or maternal pneumococcal carriage at the time of delivery and cord plasma IgG antibody titers to PspA-family 1, PspA-family 2, and Ply (model II).

**Online Supplementary Table 4. Cox regression analysis for associations between CBMC innate dPly-induced cytokine responses and age of pneumococcal colonization in 1^st^ month of life in PNG study infants**

|  | **A. Absolute cytokine levels** | | **B. Adjusted cytokine levels** | |
| --- | --- | --- | --- | --- |
| **Ply** | HR (95% CI) | P-value | HR (95% CI) | P-value |
| IL-6 | 0.45 (0.06 – 3.66) | 0.453 | 1.32 (0.96 – 1.81) | 0.085 |
| IL-10 | 1.62 (0.41 – 0.64) | 0.489 | 0.61 (0.32 – 1.17) | 0.137 |
| IFN-γ | 0.86 (0.44 – 1.69) | 0.657 | 1.22 (0.63 – 2.36) | 0.560 |
| TNF-α | 0.72 (0.33 – 1.58) | 0.408 | 1.06 (0.45 – 2.47) | 0.897 |

CBMC of PNG infants were stimulated with dPly for 24 hours to assess innate immune responses. Multivariate Cox regression models were applied to study independent associations between cord blood innate dPly cytokine responses (^10^log-transformed; pg/ml) and age of first pneumococcal upper respiratory tract colonization in the first month of life, adjusted for maternal pneumococcal carriage at the time of delivery and cord plasma dPly-specific IgG antibody titres. Associations were studied for A) absolute cytokine concentrations measured, and B) concentrations adjusted for baseline levels present in non-stimulated cultures.
